# Evidence of recent interkingdom horizontal gene transfer between bacteria and *Candida parapsilosis*

**DOI:** 10.1186/1471-2148-8-181

**Published:** 2008-06-24

**Authors:** David A Fitzpatrick, Mary E Logue, Geraldine Butler

**Affiliations:** 1 School of Biomolecular and Biomedical Science, Conway Institute, University College, Dublin, Belfield, Dublin 4, Ireland

## Abstract

**Background:**

To date very few incidences of interdomain gene transfer into fungi have been identified. Here, we used the emerging genome sequences of *Candida albicans *WO-1, *Candida tropicalis, Candida parapsilosis, Clavispora lusitaniae, Pichia guilliermondii*, and *Lodderomyces elongisporus *to identify recent interdomain HGT events. We refer to these as CTG species because they translate the CTG codon as serine rather than leucine, and share a recent common ancestor.

**Results:**

Phylogenetic and syntenic information infer that two *C. parapsilosis *genes originate from bacterial sources. One encodes a putative proline racemase (PR). Phylogenetic analysis also infers that there were independent transfers of bacterial PR enzymes into members of the Pezizomycotina, and protists. The second HGT gene in *C. parapsilosis *belongs to the phenazine F (PhzF) superfamily. Most CTG species also contain a fungal PhzF homolog. Our phylogeny suggests that the CTG homolog originated from an ancient HGT event, from a member of the proteobacteria. An analysis of synteny suggests that *C. parapsilosis *has lost the endogenous fungal form of PhzF, and subsequently reacquired it from a proteobacterial source. There is evidence that *Schizosaccharomyces pombe *and Basidiomycotina also obtained a PhzF homolog through HGT.

**Conclusion:**

Our search revealed two instances of well-supported HGT from bacteria into the CTG clade, both specific to *C. parapsilosis*. Therefore, while recent interkingdom gene transfer has taken place in the CTG lineage, its occurrence is rare. However, our analysis will not detect ancient gene transfers, and we may have underestimated the global extent of HGT into CTG species.

## Background

Lateral or horizontal gene transfer (HGT) is defined as the exchange of genes between different strains or species [1]. HGT introduces new genes into a recipient genome that are either homologous to existing genes, or belong to entirely new sequence families. Large-scale genomic sequencing of prokaryotes has revealed that gene transfer is an important evolutionary mechanism for these organisms [2,3]. HGT has been linked to the acquisition of drug resistance by benign bacteria [4], and also to the gain of genes that confer the ability to catabolize certain amino acids that are important virulence factors [5]. However there is much debate as to whether lateral gene transfer is an ubiquitous influence throughout prokaryotic genome evolution [6]. Until recently, the process of gene transfer has been assumed to be of limited significance to eukaryotes [7]. The availability of diverse eukaryotic genome sequence data is dramatically changing our views on the important role gene transfer can play in eukaryotic evolution.

The rapid increase in fungal sequence data has promoted this kingdom to the forefront of comparative genomics [8]. Whereas there is some documented evidence for HGT between fungal species [9-17] or from bacteria to fungi [18-28] [see additional file 1], overall very few incidences have been identified. There are two possible explanations: either gene transfer is indeed extremely rare amongst fungi, or it has not yet been thoroughly studied. To address this question we investigated the frequency of successful recent interdomain HGT events between prokaryotes and yeast species belonging to the CTG clade. We chose this course of action as we expect recent interdomain HGT events to be more readily identified and supported than more ancient transfers.

For the purposes of this study, we define CTG species as the immediate relatives of *C. albicans*, including *C. tropicalis, C. parapsilosis, Clavispora lusitaniae, Pichia guilliermondii*, and *Lodderomyces elongisporus*. These species have been completely sequenced, share a relatively recent common ancestor [29], and the codon CUG is translated as serine rather than leucine [30].

We used syntenic, phylogenetic and sequence based analyses to identify two cases of interdomain HGT between prokaryotes and *C. parapsilosis*, most likely involving the proteobacteria phylum. Our results suggest that extant CTG species do not readily take up exogenous DNA.

## Results and discussion

### Identification of horizontal gene transfer candidates through Blast database search

We compared all available CTG gene sets against UniProt using BlastP [31]. CTG genes with top database hits to bacterial species were identified as putative horizontally transferred genes and the resultant Blast files were inspected manually. A *D. hansenii *gene (protein ydhR precursor) with a top database hit to a bacterial sequence was not considered for further analsyes as it has previously been described [22]. After this process two genes from *C. parapsilosis *were considered for further analysis; one encodes a putative proline racemase, and the second encodes a member of the phenazine F superfamily. Related family members were identified by a second round of database searching against GenBank to ensure all available genomic data was utilized.

### Proline racemase phylogeny and characterization

**Figure 1:**
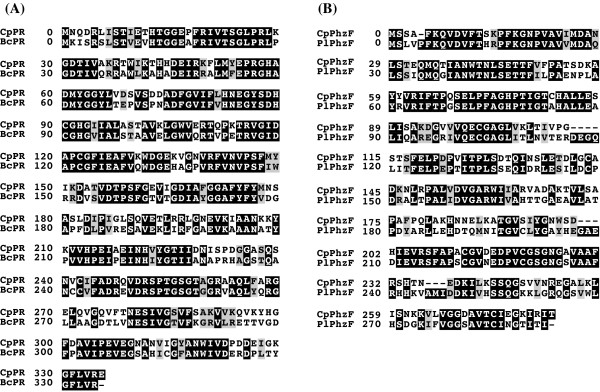
**A) **An alignment of PR proteins from *C. parapsilosis *(CpPR CPAG_02038) and *Burkholderia cenocepacia *(BcPR) was generated with MUSCLE. These proteins are 66% identical **B) **An alignment of PhzF proteins from *Candida parapsilosis *(CpPhzF CPAG_03462) and *Photorhabdus luminescens *(PlPhzF), these are 61% identical.

The *C. parapsilosis *gene (designated CPAG_02038) is most similar to a proline racemase homolog from *Burkholderia cenocepacia *AU 1054 protein (66% pairwise identity; Figure 1A). Amino acid racemases catalyze the interconversion of L- and D-amino acids by abstraction of the α-amino proton of the enzyme bound substrate [32]. CPAG_02038 lies within a large contig and is also present in a previously published genome survey of *C. parapsilosis *[33], suggesting its presence does not the result from contamination. We could not locate any related genes in any other CTG genome (using BlastP or TBlastN). Family members are widely distributed throughout the prokaryotes however, and are also located within the Pezizomycotina.

**Figure 2:**
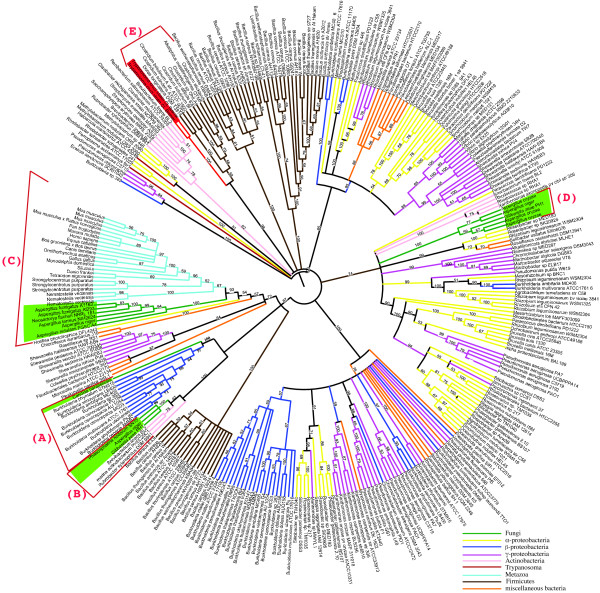
**Proline racemase maximum likelihood phylogeny**. The optimum model of protein substitution was found to be WAG+G. The number of gamma rate categories was 4 (alpha = 1.163). Bootstrap resampling (100 iterations) was undertaken and are displayed. For display purposes branches with less than 50% support were collapsed. Letters (A-E) in parentheses are used to distinguish clades and are discussed in the text. Branches are colored according to their taxonomy. Fungal branches and species names are colored green.

We extracted 321 putative proline racemases from 207 organisms, including members of the α, β, γ, and δ-proteobacteria, Actinobacteria, Fungi, Protozoa and Metazoa. Numerous species were found to have several family members [see additional file 2]; all were included for complete comparative purposes. A maximum likelihood (ML) phylogeny was reconstructed from an alignment of all the PR proteins (Figure 2).

**Figure 3:**
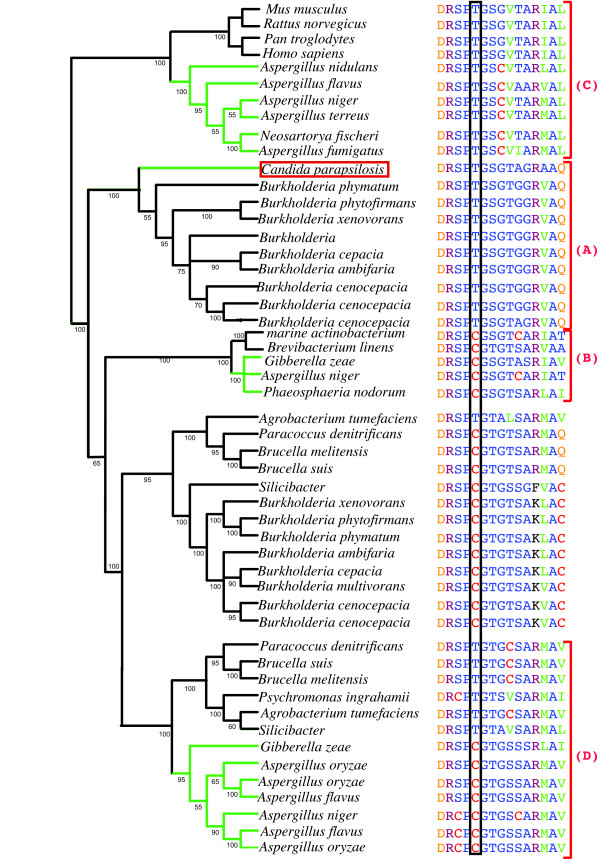
**Reduced Proline racemase maximum likelihood phylogeny with active site alignment**. Bootstrap resampling (100 iterations) was undertaken and percentages are displayed. Fungal branches are shown in green. An alignment around the active site is also displayed. Clade letters in parentheses correspond to those in Figure 2. The phylogeny is rooted around the Metazoan/Pezizomycotina specific clade (clade-C), all members of this clade have a threonine at the active site. *C. parapsilosis *and its phylogenetic neighbors have a threonine instead of a cysteine at the active site (clade-A). *A. oryzae, A. niger *and *G. zeae *all contain cysteine at the active site (clade-D). *A. flavus*, *A. oryzae*, *A. niger*, *A. nidulans *and *G. zeae *also have cysteine at the active site (clade-C).

There are a large number of polytomies displayed in Figure 2. These probably result from duplication of PR genes followed by diversifying selection, leading to a high degree of sequence heterogeneity. For example, *Agrobacterium tumefaciens *str. C58 contains three PR homologs [see additional file 2], with an average amino acid pairwise percentage identity of ~31%. *Burkholderia cenocepacia *AU 1054 contains 2 proline racemase homologs [see additional file 2], which are only 28% identical. To help resolve the evolutionary history amongst PR homologs we reconstructed an additional ML phylogeny based on a reduced dataset (Figure 3). We also reconstructed a Bayesian phylogeny using the heterogeneous CAT site model. The CAT model can account for site-specific features of sequence evolution and has been found to be more robust than other methods against phylogenetic artifacts such as long branch attraction [34]. The resultant Bayesian phylogeny is highly congruent with the ML phylogeny (not shown).

The putative *C. parapsilosis *PR homolog lies in a strongly supported (100% Bootstrap support (BP)) clade with *Burkholderia *species (Figures 2 &3 clade-A). *Burkholderia *are β-proteobacteria. However, no other β-proteobacteria, or indeed any other bacterial genus were found within clade-A (Figures 2 &3).

Although no PR homologs were identified in other CTG species, or indeed in any other of the Saccharomycotina, there are homologs in family members of the Pezizomycotina. A Pezizomycotina specific subclade is evident in our phylogeny containing *Phaeosphaeria nodorum*, *Aspergillus niger *and *Gibberella zeae *(Figures 2 &3 clade-B 100% BP). This subclade is found in a strongly supported clade with members of the Actinobacteria (Figure 2 100% BP), containing *Brevibacterium linens *and an unclassified marine actinobacterium and excluding *Rubrobacter xylanophilus *(Figure 2 87% BP). This suggests that these Pezizomycotina species obtained their PR gene from the Actinobacteridae subclass rather than the Rubrobacteridae subclass. This transfer event is another independent HGT event of a PR gene into fungi, and we hypothesize it occurred early in the Pezizomycotina lineage, as it is shared by three distantly related species. Its patchy phyletic distribution suggests it has been subsequently lost in other Pezizomycotina species.

There are also PR homologs in the Metazoans. These are found in a eukaryote clade that also contains a number of Pezizomycotina representatives (Figures 2 &3 clade-C 93% BP). Several scenarios can explain this phylogenetic positioning. Firstly, the PR gene may have been present in the last universal common ancestor of all eukaryotes but has been differentially lost in all lineages except those leading to modern day Metazoa and Pezizomycotina. Alternatively, an ancient gene transfer from bacteria to the last common ancestor (LCA) of Metazoa and Fungi could have occurred, with subsequent gene loss amongst different Metazoan and Fungal lineages. A third hypothesis is that two independent gene transfers have occurred into the Metazoan and Pezizomycotina lineages from unsampled bacterial donors. Finally, a transfer from unsampled bacteria into one of the eukaryote clades (either Metazoa or Pezizomycotina) may have occurred with subsequent transfer from one eukaryotic group to the other.

*A. niger*, *A. oryzae *and *G. zeae *all contain multiple PR homologs [see additional file 2]. One *A. niger*, one *G. zeae *and the three *A. oryzae *PR homologs are nested in a strongly supported Pezizomycotina specific subclade (Figures 2 &3 clade-D 100% BP). This subclade if found within a larger predominately proteobacterial clade (Figure 2 74% BP). This infers that there was an independent gene transfer event of a bacterial PR homolog into an ancestral Pezizomycotina species.

The phylogenetic position of the *C. parapsilosis *PR homolog (Figures 2 &3) resemble that described for the adenosine deaminase (ADA) gene in the *Dekkera bruxellensis *genome [21]. In that analysis, the authors suggest that *D. bruxellensis *and *Burkholderia *species received the ADA gene from a species not yet represented in the public sequence databases. Our PR phylogeny suggests a similar event may have occurred within clade-A, which contains only *C. parapsilosis *and *Burkholderia *species (Figures 2 &3). *Burkholderia *species are known to have a genomic repertoire that allows the transfer and receipt of exogenous DNA [35] and a number of studies have reported successful gene transfers into *Burkholderia *species [36,37]. It is possible therefore that there have been other successful gene transfers into this bacterial lineage.

The vast majority of amino acids found in living cells correspond to the L-stereoisomer [38]. However, D-amino acids are long known to be found in the cell walls of Gram positive and negative bacteria, where they are essential components of peptidoglycan [39]. Apart from low levels of D-amino acids derived from spontaneous racemization as a result of aging [40], it was assumed that only L-amino acid enantiomers were present in eukaryotes [41]. However, recent studies have reported the presence of numerous D-amino acids in an array of organisms, including mammals [42]. The first eukaryotic (proline) amino acid racemase has recently been described from the human pathogen *Trypanosoma cruzi *[43]. A high degree of sequence similarity was observed between the *T. cruzi *and bacterial homologs [43]. Our phylogeny infers that *T. cruzi *obtained its PR homolog through interdomain HGT from a member of the Firmicutes (subclass Clostridia), as it is grouped beside members of this group with a high degree of support (Figure 2 clade-E 96% BP). We performed database searches [44], against other Protozoan genomes including *Trypanosoma brucei*, *Trypanosoma congolense *and *Trypanosoma annulata*. We failed to locate a homolog in all species except for *T. vivax*.

Previous analysis has shown that *T. cruzi *and *T. vivax *are not each others closest phylogenetic neighbors, relative to the other species sampled [45]. This suggests an ancestral Trypanosoma gained the PR gene and multiple losses in different Trypanosoma lineages has subsequently occurred.

### Gene order around PR homologs

**Figure 4:**
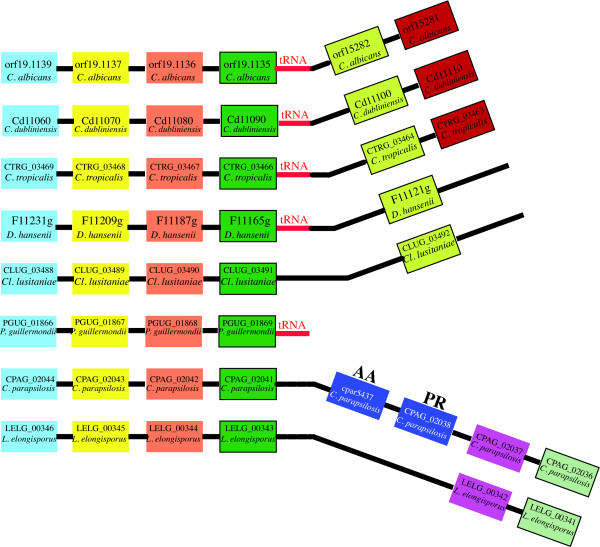
**Gene order around *C. parapsilosis *proline racemase gene**. Species names and identifiers are shown in each box. Gene identifiers relate to annotations from the Broad Institute [66]. On the left hand side orthologous genes are stacked under one another in pillars. Relative positions of t-RNA genes are shown and may indicate a breakpoint. After the breakpoint, synteny is conserved between *C. albicans*, *C. dubliniensis*, *C. tropicalis*, *D. hansenii *and *Cl. lusitaniae*. Synteny between *C. parapsilosis *and *L. elongisporus *is conserved but differs to the other CTG species. *C. parapsilosis *has a proline racemase (PR CPAG_02038) and a neutral amino acid transporter (AA cpar5437) insertion in this region. cpar5437 is absent from the Broad gene list but present in our manual gene call.

The *C. parapsilosis *PR homolog lies close to an ortholog (CPAG_02041) of orf19.1135 from *C. albicans *(Figure 4). The gene order to the left of this ORF is conserved in all CTG species, the order to the right is conserved in most CTG species apart from *C. parapsilosis *and *L. elongisporus*. *C. parapsilosis *and *L. elongisporus *are closely related [29], and an examination of synteny suggests that the PR gene (together with a second ORF, cpar5437) were inserted between CPAG_2041 and CPAG_2037 (Figure 4). cpar5437 encodes a neutral amino acid (AA) transporter. The presence of an AA transporter beside the PR homolog is interesting. If the putative proline racemase has a role in amino acid metabolism, then the presence of the transporter may be the result of an adaptive translocation to enhance the activity of the PR gene. Unlike the PR ORF the AA transporter is fungal in origin. Most CTG species contain a single neutral AA transporter; however *C. parapsilosis *and *D. hansenii *have four.

We located tRNA genes for nearly all CTG species beside the large conserved syntenic block (Figure 4). It has been shown that tRNA genes are associated with genomic breakpoints [46]. We hypothesize that a genomic rearrangement has occurred at this site in the LCA of *C. parapsilosis *and *L. elongisporus*. We cannot determine if the bacterial PR homolog was inserted into the LCA of *L. elongisporus*/*C. parapsilosis *and subsequently lost in *L. elongisporus*, or gained by *C. parapsilosis *after speciation.

We also investigated the gene order around the Pezizomycotina PR homologs [see additional file 3]. Gene synteny around the PR homologs found in clade-D (Figures 2 &3) is not conserved (not shown). Interestingly however, both *A. niger *and *G. zeae *in clade-D (Figures 2 &3) have genes containing a FAD dependent oxidoreductase domain in close proximity to their PR homologs (not shown). According to Pfam [47], FAD dependent oxidases include D-amino acid oxidases, that catalyze the oxidation of neutral and basic D-amino acids into their corresponding keto acids. The presence of these oxidases may be another example of an adaptive translocation to enhance the activity of the PR gene in these Pezizomycotina species.

*A. oryzae *has three PR homologs (Figures 2 &3 clade-C). All of these have orthologs in its close relative *A. flavus *(Figure 3 clade-C), and synteny around these is conserved [see additional file 3 clade-D]. The remaining two species in clade-C are *A. niger *and *G. zeae*. There is no evidence of conserved gene order within these species, or with *A. oryzae *or *A. flavus*. Gene order around the *A. flavus *and *A. terreus *PR homologs found in the Metazoan/Pezizomycotina clade (Figures 2 &3) is also conserved [see additional file 3], as is the order between *A. fumigatus *and *N. fishceri *[see additional file 3]. We could not locate amino acid transporters or FAD dependent oxidases beside any of the PR homologs found in clades B or C (Figure 2).

### Proline racemase codon usage

It has been shown that recently acquired genes often display an atypical codon preference when compared to other genes in the genome [48,49]. However, the transferred PR homologs have a codon usage consistent with the rest of their genomes [see additional file 4]. We undertook an analysis of variation in synonymous codon usage on all PR genes shown in Figure 2. Homologs from related species cluster together [see additional file 5]. For example, the Actinobactria, the Firmicutes and the *Burkholderia *species all inhabit unique areas in two dimensional correspondence analysis space [see additional file 5].

The majority of fungal and Metazoan PRs are clustered together [see additional file 5]. The *C. parapsilosis *PR homolog has a codon usage distinct from the other Pezizomycotina fungal PR homologs [see additional file 5], which is unsurprising as *C. parapsilosis *belongs to the Saccharomycotina subphylum. The *C. parapsilosis *homolog is also separate from the *Burkholderia *(β-proteobacteria) genes with which it forms a closely related phylogenetic group (Figures 2 &3). This suggests that the gene may have originated from a genome with no other close relatives among the species analyzed here.

### Proline racemase activity

The PR active site from *Trypanosoma cruzi, Clostridium sticklandii, Agrobacterium tumefaciens*, *Brucella melitensis *and *Pseudomonas aeruginosa *all contain cysteine at amino acid position 330 [43,50]. This amino acid is essential for enzymatic function, because substitution with serine abolishes activity [41]. However, PR homologs from human, mouse, *Rhizobium *and *Brucella *contain a threonine instead of a cysteine at position 330 [41]. We observed that cysteine is found in the equivalent position in many of the bacterial proteins. The Pezizomycotina PR genes found in clade-B and clade-D contain a cysteine at the active site (Figure 3). The PR homologs found in the Metazoan/Pezizomycotina clade (clade-B) have a threonine at position 330. Similarly, the *C. parapsilosis *PR homolog, together with its relatives from *Burkholderia *all contain a threonine (Figure 3). However, *Burkholderia *species have multiple PR homologs [see additional file 2] with a cysteine as the active site (not shown). It is not clear what effect the substitution has on enzyme activity. It has been suggested that homologs containing threonine at the active site are not true PRs [41], but may instead belong to a superfamily. We cannot detect any difference in the ability of *C. parapsilosis*, the other CTG species or any of the Pezizomycotina species to utilize D-proline as growth media (data not shown). We therefore cannot confidently infer the function of the PR homologs in the fungi analyzed here.

### Phenazine F phylogeny and characterisation

The *C. parapsilosis *gene (designated CPAG_03462) is most similar to a *Photorhabdus luminescens *phenazine F (PhzF) protein with 61% pairwise identity (Figure 1B). Phenazines are biologically active compounds, all of which have a characteristic tricyclic ring system and have been shown to confer a selective growth advantage to organisms which secrete them, as they possess broad-spectrum antibiotic activity towards bacteria, fungi and higher eukaryotes [51]. In *Pseudomonas*, the best studied phenazine producer, PhzF is part of an operon required for the conversion of chorismic acid to phenazine-1-carboxylate (PCA) [52]. PhzF homologs were identified in most of the CTG species tested as well as several other fungal species. However, we could not identify a PhzF homolog in the *L. elongisporus *genome, even when multiple TBlastN and BlastN searches were used.

PhzF homologs were extracted from GenBank for subsequent phylogenetic analysis. In total 181 representative protein coding sequences distributed amongst 154 organisms were used. These taxa were distributed amongst α, β, γ and δ-proteobacteria, Actinobacteria, Fungi, Firmicutes a well as other bacterial groups.

**Figure 5:**
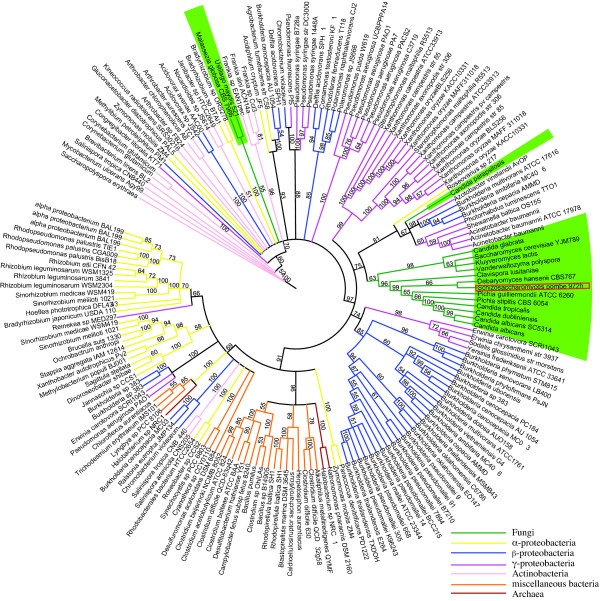
**PhzF maximum likelihood phylogeny**. The optimum model of protein substitution was found to be WAG+G. The number of gamma rate categories was 4 (alpha = 0.873). Bootstrap resampling (100 iterations) was undertaken and are displayed. For display purposes branches with less than 50% support were collapsed. Branches are colored according to their taxonomy. Fungal branches are shown in green. The *S. pombe *PhzF homolog is highlighted with a red rectangle.

We aligned all sequences and reconstructed a PhzF ML phylogeny (Figure 5). The *C. parapsilosis *PhzF homolog is found in a clade with members of the β-proteobacteria *(Burkholderia multiovorans, Burkholderia cepacia, Burkholderia ambifaria*), α-proteobacteria (*Roseovarius*) and the γ-proteobacteria (*Azotobacter vinelandii, Acinetobacter baumannii, Shewanella baltica and Photorhabdus luminescens*) (81% BP). In contrast, all other PhzF homologs from CTG species are in a completely separate clade (Figure 5). These form a sister group (63% BP) to PhzF homologs from other Saccharomycotina species (*C. glabrata, Saccharomyces cerevisiae, Kluyveromyces lactis *and *Vanderwaltozyma polyspora*). All three clades are grouped together in a larger clade with high support (75% BP).

The sister group relationship between the PhzF homologs from the Ascomycota and the proteobacteria clade is intriguing (Figure 5), as it suggests that an ancestral Saccharomycotina species gained the PhzF homolog from a proteobacteria. The bacterial PhzF gene has subsequently been retained after multiple speciation events, but lost in *C. parapsilosis*. We hypothesize that *C. parapsilosis *has recently reacquired a bacterial PhzF homolog from a proteobacterial source, as it is grouped (81% BP) within a proteobacterial subclade. To test this hypothesis we reconstructed constrained trees that placed *C. parapsilosis *together with the remaining Ascomycota species [see additional file 6 C-H]. The AU test of phylogenetic tree selection [53], showed that the original unconstrained tree (groups *C. parapsilosis *with proteobacteria) receives the optimal likelihood tree score, and the differences in likelihood scores when compared to the constrained trees [see additional file 6], are significant (P < 0.05). This is also supported by spectral analysis [see additional file 7].

Our phylogeny shows that the *Schizosaccharomyces pombe *PhzF homolog is found in a clade containing all CTG PhzF homologs (Figure 5 99% BP). Furthermore it is grouped beside *D. hansenii *(66% BP). *S. pombe *is not a member of the Saccharomycotina, it belongs to the Taphrinomycotina subphylum. The genome sequences of *Schizosaccharomyces japonicus *and *Schizosaccharomyces octosporus *have recently been completed [54]. We could not locate a PhzF homolog in *S. japonicus *but did locate a homolog in *S. octosporus *using a TBlastN search strategy. Phylogenetic analysis has shown that *S. pombe *and *S. octosporus *are more closely related to one another than to *S. japonicus *[55]. Therefore we hypothesize that the LCA ancestor of *S. pombe *and *S. octosporus *gained the PhzF gene from an ancestral *D. hansenii*-like species after speciation from *S. japonicus*. We reconstructed a constrained tree that placed *S. pombe *outside the Saccharomycotina clade [see additional file 6B]. The approximately unbiased test of phylogenetic tree selection (AU test) [53], showed that the phylogenetic inferences of the unconstrained tree are significantly better (P < 0.05) than the constrained tree [see additional file 6]. This infers that *S. pombe *has obtained a PhzF homolog from a member of the CTG clade.

A small basidiomycete clade is evident amongst prokaryote species (Figure 5). Both *Ustilago maydis *and *Malassezia globosa *belong to the Ustilaginomycotina subphylum. Therefore our phylogeny infers that an ancestral Ustilaginomycotina species gained a PhzF gene from an unknown bacterial source, and both species have retained this after speciation.

A correspondence analysis of synonymous codon usage for all PhzF homologs was also performed and is shown in additional information [see additional file 8]. The *S. pombe *PhzF homolog has a codon usage pattern very similar to the *D. hansenii *protein.

### Gene order around PhzF

**Figure 6:**
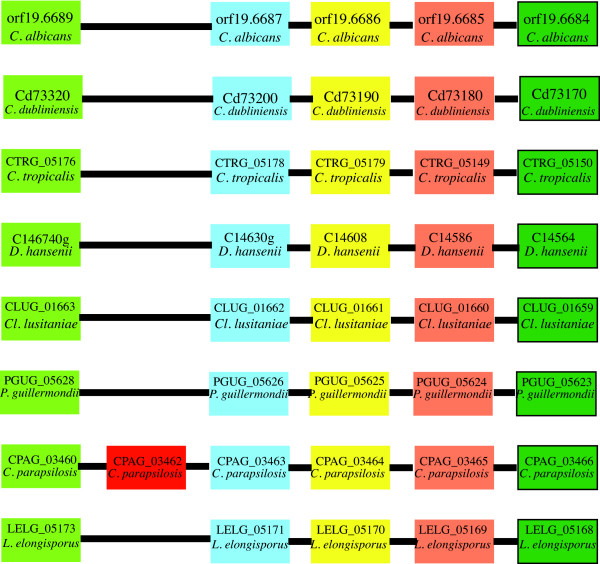
**Gene order around *C. parapsilosis *PhzF gene**. Species names and gene identifiers are shown in each box. Orthologous genes are stacked under one another in pillars. The *C. parapsilosis *PhzF homolog (CPAG_03462) is highlighted with a red box. Synteny relative to the *C. parapsilosis *PhzF homolog is conserved in all species. Other CTG PhzF homologs are found in completely different regions of the genome relative to *C. parapsilosis*. *L. elongisporus *is the only CTG species missing a PhzF gene, and there is no evidence for a pseudogene in the genome.

Analysis of the genes adjacent to the PhzF homolog in *C. parapsilosis *shows that there is a high conservation of gene synteny and supports our hypothesis that PhzF was recently acquired in this species (Figure 6). Homologs in the other CTG species are located in completely different regions of the genome relative to *C. parapsilosis *(not shown). For example, the *C. albicans *PhzF homolog is located between orf19.5619 and orf19.5621, whereas the *C. parapsilosis *homolog is found between orf19.6689 & orf19.6687 relative to *C. albicans *SC5314 (Figure 6). However, the *L. elongisporus *genome contains no PhzF homolog, either at a position equivalent to the *C. parapsilosis *copy or elsewhere in the genome.

We propose that the LCA of *L. elongisporus *and *C. parapsilosis *lost the PhzF gene present in the other CTG species, and a second (new) copy was subsequently gained by *C. parapsilosis *after speciation. We have partial sequence data (unpublished) from *Candida orthopsilosis*, a species so closely related to *C. parapsilosis *that it was once designated *C. parapsilosis *group II [56]. We located a *C. orthopsilosis *PR homolog that is 83% identical (at the amino acid level) to the *C. parapsilosis *copy. This implies that the common ancestor of *C. parapsilosis *and *C. orthopsilosis *acquired the bacterial PhzF homolog after speciation from *L. elongisporus*.

Mechanisms of gene transfer into fungi are poorly understood. To date no DNA uptake mechanism has been identified in CTG species. Interkingdom conjugation between bacteria and yeast has been observed however [57-59]. Similarly, *Saccharomyces cerevisiae *has been shown to be transformant competent under certain conditions [60]. CTG species are known interact with bacteria *in vivo *[61], and it is therefore possible that interkingdom conjugation and transformation may facilitate DNA transfer in *C. parapsilosis*. These mechanisms may also be applicable to the Pezizomycotina species examined in this analysis.

## Conclusion

We investigated the frequency of recent interkingdom gene transfer between CTG and bacterial species. We located two strongly supported incidences of HGT, both within the *C. parapsilosis *genome. We also located independent transfers into the Pezizomycotina, Basidiomycotina and Protozoan lineages.

We cannot determine the exact origin of the PR homolog (CPAG_02038) found in the *C. parapsilosis *genome. However, based on its phylogenetic position it either originated from a *Burkholderia *source, or more likely an organism not yet represented in the sequence databases. Our PR phylogenetic analysis also suggests there were two independent transfers into Pezizomycotina species, one from an Actinobacterial source, and the second is from an unknown proteobacterial source. There is also evidence that *T. cruzi *has obtained its PR homolog from a Firmicutes species. The transferred PR genes analyzed here belong to a superfamily of proline racemases, although we cannot determine their exact function in the fungal species examined. Their proximity to an amino acid transporter (in *C. parapsilosis*) and a FAD dependent oxidoreductase (in *A. niger *and *G. zeae*) suggests they do have a role in amino acid metabolism. Furthermore, evidence of multiple independent transfers into fungi suggests the protein does confer a biological advantage, although we cannot determine what is. The bacteria-derived PR gene has the potential to be a novel antifungal drug target as there would be no undesired host protein-drug interactions.

The bacterial PhzF homolog (CPAG_03462) found in *C. parapsilosis *most likely originated from a proteobacterial source. Most CTG species examined contained PhzF homologs, with the exception of *L. elongisporus*. The crystal structure the PhzF homolog in *S. cerevisiae *has been determined and while its function remains unknown, it is not thought to be involved in phenazine production [62]. We postulate that the PhzF homolog present in other CTG species was initially lost by the ancestor of *C. parapsilosis *and *L. elongisporus*, but subsequently regained by *C. parapsilosis *through HGT. The loss of eukaryote genes and subsequent reacquisition of a prokaryotic copy has previously been described in yeast, and can confer specific metabolic capabilities. An analysis of the biotin biosynthesis pathway discovered that the ancestor of *Candida*, *Debaryomyces*, *Kluyveromyces *and *Saccharomyces *lost the majority of the pathway after the divergence from the ancestor of *Y. lipolytica*. However, *Saccharomyces *species have rebuilt the biotin pathway through gene duplication/neofunctionalization after horizontal gene transfer from α and γ proteobacterial sources [20]. The acquisition of the *URA1 *gene (encoding dihydroorotate dehydrogenase) from *Lactobacillus *and replacement of the endogenous gene in *S. cerevisiae*, allowed growth under anaerobic conditions [19]. Similarly, acquisition of *BDS1 *(alkyl-aryl-sulfatase) from proteobacteria may have enabled the survival of *S. cerevisiae *in a harsh soil environment [19]. Our PhzF phylogeny suggests that the PhzF homolog found in most CTG species originated from an ancient HGT event, from a member of the proteobacteria. Our analysis also shows that *S. pombe *has obtained a PhzF homolog from a CTG species, most likely one closely related to *D. hansenii*. There is also phylogenetic evidence showing that an ancestral Ustilaginomycotina species gained a PhzF gene from an unknown bacterial source. We cannot however, determine the biological advantage to the organisms.

Although it was not the major goal of this study, we did locate HGT from bacteria into fungal genomes outside the CTG clade, and also inter-fungal transfers. In a previous analysis of HGT in diplomonads, fifteen genes were found to have undergone HGT [18]. There is phylogenetic evidence that these genes have undergone independent transfers into other eukaryotic lineages including Fungi. Therfore, in eukaryotes just as HGT has affected some species more than others [63], there may be groups of genes that are more likely to be taken up through HGT than others. We cannot test this directly however, as we have not identified all cases of HGT from bacteria to fungi outside the CTG clade.

Our analysis indicates that recent interkingdom gene transfer into extant CTG species is negligible. This supports a previous hypothesis that genetic code alterations blocks horizontal gene transfer [64]. It should be noted however that we searched for recent bacterial gene transfers into individual CTG species, and not for more ancient transfers. We took this approach because the presence of recently gained bacterial genes in a eukaryote genome should be readily detected compared to older transfers. Similarly, we have not investigated eukaryote-to-eukaryote transfers. It is therefore possible that we have underestimated the overall rate of HGT into the CTG lineage. The discovery of HGT in other fungal lineages implies that HGT plays an important role in fungal evolution and deserves further analysis. In particular a strategy which can detect ancient gene transfers would be meaningful.

## Methods

### Sequence data

The complete *C. albicans *(SC5314) genome (Assembly 19) was obtained from the *Candida *genome database [65]. The Broad institute have sequenced and annotated five CTG species (*C. albicans *(WO-1), *C. tropicalis*, *L. elongisporus*, *P. guilliermondii*, and *Cl. lusitaniae*). These genomes were obtained directly from the Broad Institute [66]. Gene sets for the *C. dubliniensis *were downloaded from GeneDB [44].

The incomplete *C. parapsilosis *geneome was downloaded from the Sanger Institute [67]. Gene annotations were performed using two separate approaches. The first involved a reciprocal best BLAST [31] search with a cutoff E- value of 10^-7 ^of *Candida albicans *SC5314 protein coding genes against the unannotated *C. parapsilosis *genome. Top BLAST hits longer than 300 nucleotides were retained as putative open reading frames. The second approach involved a pipeline of analysis that combined several different gene prediction programs, including *ab initio *programs SNAP [68], Genezilla [69], and AUGUSTUS [69], with gene models from Exonerate [70] and Genewise [71] based on alignments of proteins and Expressed Sequence Tags. Putative gene sets from both approaches were imported into Artemis [72] and cross corroborated manually. The resultant gene sets contained 5,823 protein-coding genes. The *C. parapsilosis *genome was also annotated by the Broad Institute, and where possible we have used the gene names they assigned.

The UniProt database (v11.1) was downloaded [73]. Database searches against GenBank refer to release 164.0.

### Blast based approach to detect potential horizontally transferred genes

Taking one CTG species at a time, we located gene families of interest by comparing individual protein coding genes against the UniProt database (v11.1) using the BlastP algorithm [31] with a cutoff expectation (E) value of 10^-20^. To use all available sequence data, CTG proteins with a top database hit to a bacterial protein in UniProt were extracted for a second round of database searching against GenBank (E value of 10^-20^). Proteins which also had a top database hit to a bacterial protein in GenBank were considered as possible incidences of horizontal gene transfer. All putative homologs were extracted from GenBank and searched against the relevant CTG genome to ensure a reciprocal best Blast hit. For completeness, CTG proteins not yet deposited in GenBank were added to gene families of interest where appropriate.

Accession numbers for all sequences used in this analysis can be found in additional material [see additional file 2].

### Phylogenetic methods

Gene families were aligned using MUSCLE (v3.6) [74] using the default settings. Obvious alignment ambiguities were corrected manually.

Phylogenetic relationships were inferred using maximum likelihood methods. Appropriate protein models of substitution were selected for each gene family using ModelGenerator [75]. One hundred bootstrap replicates were then carried out with the appropriate protein model using the software program PHYML [76] and summarized using the majority-rule consensus method.

We performed the approximately unbiased test of phylogenetic tree selection [53], to assess whether differences in topology between constrained and unconstrained gene trees are no greater than expected by chance.

### Codon usage analysis and spectral analysis

To determine if the putative HGT genes had a different codon usage pattern to the host genome an analysis of variation in synonymous codon usage was undertaken using the GCUA software [77]. Individual correspondence analyses of raw codon counts for the *Candida parapsilosis*, *Ustilago maydis, Malassezia globosa, Aspergillus flavus, Aspergillus niger, Gibberella zeae, Aspergillus oryzae, Phaeosphaeria nodorum*, and *Schizosaccharomyces pombe *genomes were performed, with the first four principal axes being used to evaluate synonymous codon usage patterns. Similar analyses were also carried out on members of the proline racemase and phenazine F gene families displayed in Figures 2 and 4. We used spectrum [78] to perform a spectral analysis on a subset of the phenazine data.

## Authors' contributions

DAF, MEL and GB were involved in the design phase. MEL predicted genes in unannotated genomes. DAF sourced homologs, examined synteny and performed phylogenetic analyses. DAF and GB drafted the manuscript. All authors read and approved the final manuscript.

## Supplementary Material

Additional file 1 **Examples of reported incidences of interkingdom gene transfer between prokaryotes and fungi**. One *Kluyveromyces lactis *gene (KLLA0D19949g) previously highlighted [22], been omitted as it is no longer recognized as an ORF. *Y. lipolytica *genes denoted with a * and ^ indicate possible gene duplications after HGT. Click here for file

Additional file 2 **GenBank accession numbers for PR (A) and PhzF (B) sequences used in this analysis**. Species identified with an * use the accession numbers created by the Broad Institute [66] or the Wellcome Trust Sanger Institute [67]. Click here for file

Additional file 3 **Gene order around Pezizomycotina proline racemase genes**. Species names and identifiers are shown in each box. PR genes are labeled. Gene identifiers relate to annotations from the Broad Institute. Clade letters in parentheses correspond to those in Figure 2. There is evidence for conserved gene synteny between some species such as *A. oryzae *and *A. flavus *(clade-C). *A. flavus*/*A. terreus *and *N. fischeri*/*A. fumigatus *in the Metazoan/Pezizomycotina clade (B). The *A. flavus *gene denoted by a * is absent from the Broad gene set but we were able to locate it with a BlastX search. Click here for file

Additional file 4 **Correspondence analysis of codon usage**. Correspondence analysis of codon usage in the *C. parapsilosis *(1), *U. maydis *(2), *M. globosa *(3), *A. flavus *(4), *A. niger *(5), *G. zeae *(6), *A. oryzae *(7), *P. nodorum *(8), and *S. pombe *(9) genomes. Transferred genes are highlighted. All have a codon usage similar to the rest of their genomes which is unsurprising as transferred genes have been shown to ameliorate their codon usage to their hosts [79]. Click here for file

Additional file 5 **Correspondence analysis of codon usage in the proline racemase gene family analyzed in this study**. Major groups are color-coded. The *C. parapsilosis *PR gene has a codon usage pattern distinct from other fungal species in this analysis. It is also quite distinct from the *Burkholderia *(β-proteobacteria) species, which were found to be its phylogenetic neighbors. Click here for file

Additional file 6 **Trees for approximately unbiased test for PhzF homologs**. Tree A is the original unconstrained topology, which groups *C. parapsilosis *with proteobacteria. Topology B is a constrained tree that places S. pombe outside the Saccharomycotina clade. Topologies C-H are constrained and place *C. parapsilosis *amongst the other Saccharomycotina species. Log likelihood scores for each tree are given. To assess the likelihood that any differences in topology between the inferred trees is no more significant that that expected by chance, we performed the approximately unbiased test. The AU test shows that the unconstrained tree receives the optimal likelihood tree score. Furthermore, the differences in likelihood scores when compared to the constrained trees are significant (P < 0.05). Therefore based on these results the placement of the *C. parapsilosis *homolog in the proteobacterial clade to the exclusion of the Saccharomycotina and S. pombe within the Saccharomycotina clade is significant. Click here for file

Additional file 7 **PhzF spectral analysis**. Analysis was performed on the Saccharomycotina and selected proteobacterial clade. Bars above the x-axis represent frequency of support for each split. Bars below the x-axis represent the sum of all corresponding conflicts. Clad grams above columns represent the corresponding splits in the data. There is no support for the placement of *C. parapsilosis *with the other Saccharomycotina species. Click here for file

Additional file 8 **Correspondence analysis of codon usage in the PhzF gene family analyzed in this study**. Major groups are color-coded. The *C. parapsilosis *PhzF gene has a codon usage pattern similar to other CTG species analyzed. It is quite distinct from the proteobacterial species that were found to be its phylogenetic neighbors. Click here for file
