# Supplementary material for: Evidence of recent interkingdom horizontal gene transfer between bacteria and Candida parapsilosis
Source: BMC Evol Biol. 2008 Jun 24;8:181. doi: 10.1186/1471-2148-8-181 (PMC2459174; doi:10.1186/1471-2148-8-181)
Supplement: Additional file 1 — Examples of reported incidences of interkingdom gene transfer between prokaryotes and fungi. One Kluyveromyces lactis gene (KLLA0D19949g) previously highlighted [22], been omitted as it is no longer recognized as an ORF. Y. lipolytica genes denoted with a * and ^ indicate possible gene duplications after HGT. [file 1471-2148-8-181-S1.doc]

| **Recipient species** | **Function** | **Best bacterial Blast hit** | **Reference** |
| --- | --- | --- | --- |
| Ascomycota species | Class III Alcohol Dehydrogenase | α-proteobacteria | [18] |
| Ascomycota species | Flavohemoglobin | Proteobacteria | [18] |
| *Saccharomyces cerevisiae* | Dihydroorotate dehydrogenase | *Lactococcus lactis* | [19, 23] |
| *Saccharomyces cerevisiae* | Alkyl-aryl-sulfatase | *Rhodopseudomonas palustris* | [19] |
| *Saccharomyces cerevisiae* | Nucleoside-sugar epimerase | *Bacillus coagulans* | [19] |
| *Saccharomyces cerevisiae* | 3 unknown proteins |  | [19] |
| *Saccharomyces cerevisiae* | Galactoside acetyl-transferase | *Methanosarcina mazei* | [19] |
| *Saccharomyces cerevisiae* | Methionine gamma lyase | *Yersinia pestis* | [19] |
| *Saccharomyces cerevisiae* | 7,8-diamino pelargonic acid synthetase | *Yersinia bercovieri* | [19, 20] |
| *Saccharomyces cerevisiae* | Dethiobiotin synthetase | *Gluconobacter oxydans* | [19, 20] |
| *Saccharomyces cerevisiae* | Glutathione S-transferase | *Bradyrhizobium japonicum* | [20] |
| *Saccharomyces cerevisiae* | Oxidoreductase | *Ralstonia solanacearum* | [20] |
| *Saccharomyces cerevisiae* | Acetyl-transferase | *Enterococcus faecalis* | [20] |
| *Dekkera bruxellensis* | Adenosine deaminase | *Burkholderia cenocepacia* | [21] |
| *Kluyveromyces lactis* | Conserved glyoxalase domain protein | *Xanthomonas axonopodis* | [22] |
| *Kluyveromyces lactis* | Alcohol dehydrogenase | *Neisseria meningitidis* | [22] |
| *Kluyveromyces lactis* | Hypothetical protein | *Shewanella oneidensis* | [22] |
| *Kluyveromyces lactis* | negative regulatory protein, aacetyl transferase domain | *Bacillus subtilis* | [22] |
| *Debaryomyces hansenii* | ydhR precursor | *Bacillus cereus* | [22] |
| *Yarrowia lipolytica* | D-aminopeptidase | *Rhizobium loti* | [22] |
| *Yarrowia lipolytica* | 3 Hypothetical proteins |  | [22] |
| *Yarrowia lipolytica* | Adenylate kinase | *Bacillus subtilis* | [22] |
| *Yarrowia lipolytica ** | Putative acetyltransferase | *Rhizobium meliloti* | [22] |
| *Yarrowia lipolytica ** | Putative acetyltransferase | *Rhizobium meliloti* | [22] |
| *Yarrowia lipolytica* ^ | Yee/YedE family protein | *Pseudomonas putida* | [22] |
| *Yarrowia lipolytica* ^ | Yee/YedE family protein | *Pseudomonas putida* | [22] |
| Ascomycete species | Histidine kinases | *Streptomyces coelicolor* | [24] |
| *Nectria haematococca* | *PEP* cluster of disease genes |  | [25] |
| Ascomycete species | β-glucuronidase | High GC gram+ bacteria | [26] |
| *Orpinomyces joyonii* | endoglucanase | *Fibrobacter succinogenes* | [27] |
| *Aspergillus* species | Group II Catalases | Unknown bacterial source | [28] |
