# Supplementary material for: Evidence of recent interkingdom horizontal gene transfer between bacteria and Candida parapsilosis
Source: BMC Evol Biol. 2008 Jun 24;8:181. doi: 10.1186/1471-2148-8-181 (PMC2459174; doi:10.1186/1471-2148-8-181)
Supplement: Additional file 2 — GenBank accession numbers for PR (A) and PhzF (B) sequences used in this analysis. Species identified with an * use the accession numbers created by the Broad Institute [66] or the Wellcome Trust Sanger Institute [67]. [file 1471-2148-8-181-S2.doc]

**(A)**

| alpha proteobacterium BAL199 | ZP_02188341.1 |
| --- | --- |
| alpha proteobacterium HTCC2255 | ZP_01446623.1 |
| Acinetobacter baumannii | CAM87235.1 |
| Acinetobacter baumannii ATCC 17978 | YP_001084355.1 |
| Agrobacterium tumefaciens str. C58 | NP_353428.1 |
| Agrobacterium tumefaciens str. C58 | NP_355981.1 |
| Agrobacterium tumefaciens str. C58 | NP_356688.1 |
| Algoriphagus sp. PR1 | ZP_01718981.1 |
| Alkalilimnicola ehrlichei MLHE-1 | YP_742890.1 |
| Alkaliphilus metalliredigens QYMF | YP_001318555.1 |
| Alkaliphilus oremlandii OhILAs | YP_001511599.1 |
| Alteromonadales bacterium TW-7 | ZP_01613064.1 |
| Aspergillus flavus * | AFL2G_11282.2 |
| Aspergillus flavus* | AFL2G_11333.2 |
| Aspergillus flavus* | AFL2G_01750.2 |
| Aspergillus fumigatus A1163 | EDP51050.1 |
| Aspergillus fumigatus Af293* | Afu5g02000 |
| Aspergillus nidulans FGSC A4* | AN2121.2 |
| Aspergillus niger* | fge1_pm_C_24000003 |
| Aspergillus niger* | e_gw1_3.2327 |
| Aspergillus niger* | e_gw1_12.538 |
| Aspergillus oryzae* | AO090010000012 |
| Aspergillus oryzae* | AO090010000067 |
| Aspergillus oryzae* | AO090103000370 |
| Aspergillus terreus NIH2624* | ATEG_09819.1 |
| Aurantimonas sp. SI85-9A1 | ZP_01228511.1 |
| Bacillus anthracis str. A2012 | ZP_00393085.1 |
| Bacillus anthracis str. Ames | NP_843412.1 |
| Bacillus anthracis str. Ames | NP_845179.1 |
| Bacillus anthracis str. Ames | NP_845180.1 |
| Bacillus cereus 03BB108 | ZP_02601338.1 |
| Bacillus cereus AH1134 | ZP_02524410.1 |
| Bacillus cereus AH1134 | ZP_02524411.1 |
| Bacillus cereus AH1134 | ZP_02526630.1 |
| Bacillus cereus AH187 | ZP_02254232.1 |
| Bacillus cereus AH187 | ZP_02255378.1 |
| Bacillus cereus AH187 | ZP_02255379.1 |
| Bacillus cereus AH820 | ZP_02259911.1 |
| Bacillus cereus AH820 | ZP_02259912.1 |
| Bacillus cereus AH820 | ZP_02261048.1 |
| Bacillus cereus ATCC 10987 | NP_977317.1 |
| Bacillus cereus ATCC 10987 | NP_979166.1 |
| Bacillus cereus ATCC 10987 | NP_979167.1 |
| Bacillus cereus ATCC 14579 | NP_830691.1 |
| Bacillus cereus ATCC 14579 | NP_832584.1 |
| Bacillus cereus ATCC 14579 | NP_832585.1 |
| Bacillus cereus B4264 | ZP_02577142.1 |
| Bacillus cereus B4264 | ZP_02577143.1 |
| Bacillus cereus E33L | YP_082406.1 |
| Bacillus cereus E33L | YP_084147.1 |
| Bacillus cereus E33L | YP_084148.1 |
| Bacillus cereus G9241 | ZP_00235895.1 |
| Bacillus cereus G9241 | ZP_00238358.1 |
| Bacillus cereus G9241 | ZP_00238359.1 |
| Bacillus cereus G9842 | ZP_02582882.1 |
| Bacillus cereus G9842 | ZP_02582883.1 |
| Bacillus cereus G9842 | ZP_02584624.1 |
| Bacillus cereus H3081.97 | ZP_02596055.1 |
| Bacillus cereus NVH0597-99 | ZP_02588328.1 |
| Bacillus cereus NVH0597-99 | ZP_02593112.1 |
| Bacillus cereus W | ZP_02538984.1 |
| Bacillus cereus W | ZP_02541876.1 |
| Bacillus sp. B14905 | ZP_01723674.1 |
| Bacillus thuringiensis serovar israelensis ATCC 35646 | ZP_00742539.1 |
| Bacillus thuringiensis serovar israelensis ATCC 35646 | ZP_00742540.1 |
| Bacillus thuringiensis serovar konkukian str. 97-27 | YP_035142.1 |
| Bacillus thuringiensis serovar konkukian str. 97-27 | YP_036918.1 |
| Bacillus thuringiensis serovar konkukian str. 97-27 | YP_036919.1 |
| Bacillus thuringiensis str. Al Hakam | YP_893693.1 |
| Bacillus thuringiensis str. Al Hakam | YP_895336.1 |
| Bacillus thuringiensis str. Al Hakam | YP_895337.1 |
| Bacillus weihenstephanensis KBAB4 | YP_001643688.1 |
| Bacillus weihenstephanensis KBAB4 | YP_001645466.1 |
| Bacillus weihenstephanensis KBAB4 | YP_001645467.1 |
| Blastopirellula marina DSM 3645 | ZP_01091706.1 |
| Bordetella petrii DSM 12804 | YP_001629866.1 |
| Bos taurus | NP_001029558.1 |
| Brevibacterium linens BL2 | ZP_00378706.1 |
| Brevibacterium linens BL2 | ZP_00380483.1 |
| Brevibacterium linens BL2 | ZP_00381268.1 |
| Brevibacterium linens BL2 | ZP_00381269.1 |
| Brucella abortus biovar 1 str. 9-941 | YP_222451.1 |
| Brucella canis ATCC 23365 | YP_001592207.1 |
| Brucella melitensis 16M | NP_539175.1 |
| Brucella melitensis 16M | NP_540503.1 |
| Brucella ovis ATCC 25840 | YP_001258374.1 |
| Brucella suis 1330 | NP_697371.1 |
| Brucella suis 1330 | NP_698773.1 |
| Brucella suis ATCC 23445 | YP_001623023.1 |
| Burkholderia ambifaria AMMD | YP_775438.1 |
| Burkholderia ambifaria AMMD | YP_775657.1 |
| Burkholderia ambifaria MC40-6 | ZP_01553263.1 |
| Burkholderia ambifaria MC40-6 | ZP_01553997.1 |
| Burkholderia ambifaria MC40-6 | ZP_01555905.1 |
| Burkholderia ambifaria MC40-6 | ZP_01555908.1 |
| Burkholderia cenocepacia AU 1054 | YP_623869.1 |
| Burkholderia cenocepacia AU 1054 | YP_624085.1 |
| Burkholderia cenocepacia MC0-3 | ZP_01561220.1 |
| Burkholderia cenocepacia MC0-3 | ZP_01564482.1 |
| Burkholderia cenocepacia PC184 | ZP_00979241.1 |
| Burkholderia dolosa AUO158 | EAY70346.1 |
| Burkholderia dolosa AUO158 | ZP_00984757.1 |
| Burkholderia mallei ATCC 23344 | YP_106025.1 |
| Burkholderia mallei GB8 horse 4 | ZP_00442095.1 |
| Burkholderia multivorans ATCC 17616 | YP_001584233.1 |
| Burkholderia multivorans ATCC 17616 | YP_001584419.1 |
| Burkholderia multivorans ATCC 17616 | YP_001585227.1 |
| Burkholderia multivorans ATCC 17616 | YP_001585230.1 |
| Burkholderia oklahomensis EO147 | ZP_02360067.1 |
| Burkholderia phymatum STM815 | ZP_01503631.1 |
| Burkholderia phymatum STM815 | ZP_01506301.1 |
| Burkholderia phytofirmans PsJN | ZP_01506930.1 |
| Burkholderia phytofirmans PsJN | ZP_01508905.1 |
| Burkholderia pseudomallei 14 | ZP_02414158.1 |
| Burkholderia pseudomallei 1710b | YP_337044.1 |
| Burkholderia pseudomallei DM98 | ZP_02405633.1 |
| Burkholderia pseudomallei DM98 | ZP_02405634.1 |
| Burkholderia pseudomallei K96243 | YP_110352.1 |
| Burkholderia sp. 383 | YP_372329.1 |
| Burkholderia sp. 383 | YP_372418.1 |
| Burkholderia sp. 383 | YP_372652.1 |
| Burkholderia thailandensis Bt4 | ZP_02385591.1 |
| Burkholderia thailandensis E264 | YP_440255.1 |
| Burkholderia thailandensis MSMB43 | ZP_02465625.1 |
| Burkholderia thailandensis TXDOH | ZP_02371740.1 |
| Burkholderia ubonensis Bu | ZP_02377206.1 |
| Burkholderia ubonensis Bu | ZP_02382444.1 |
| Burkholderia vietnamiensis G4 | YP_001117124.1 |
| Burkholderia xenovorans LB400 | YP_553895.1 |
| Burkholderia xenovorans LB400 | YP_554280.1 |
| Burkholderia xenovorans LB400 | YP_554430.1 |
| Candida parapsilosis | cpar5436 |
| Canis familiaris | XP_547837.1 |
| Cellulophaga sp. MED134 | ZP_01049413.1 |
| Chloroflexus aurantiacus J-10-fl | YP_001636552.1 |
| Chromobacterium violaceum ATCC 12472 | NP_902496.1 |
| Chromohalobacter salexigens DSM 3043 | YP_574388.1 |
| Chromohalobacter salexigens DSM 3043 | YP_574751.1 |
| Clavibacter michiganensis subsp. michiganensis NCPPB 382 | YP_001221551.1 |
| Clavibacter michiganensis subsp. sepedonicus | CAQ00193.1 |
| Clostridium botulinum A str. ATCC 3502 | YP_001254971.1 |
| Clostridium botulinum Bf | ZP_02617215.1 |
| Clostridium difficile 630 | YP_001089754.1 |
| Clostridium scindens ATCC 35704 | ZP_02432001.1 |
| Clostridium sticklandii | CAB71312.1 |
| Colwellia psychrerythraea 34H | YP_268192.1 |
| Colwellia psychrerythraea 34H | YP_268195.1 |
| Danio rerio | XP_694147.1 |
| Dorea longicatena DSM 13814 | ZP_01996167.1 |
| Equus caballus | XP_001492119.1 |
| Erwinia carotovora subsp. atroseptica SCRI1043 | YP_050790.1 |
| Ferroplasma acidarmanus fer1 | ZP_01709247.1 |
| Flavobacteriales bacterium ALC-1 | ZP_02181843.1 |
| Flavobacteriales bacterium HTCC2170 | ZP_01105902.1 |
| Flavobacteriales bacterium HTCC2170 | ZP_01106480.1 |
| Flavobacterium sp. MED217 | ZP_01060434.1 |
| Fulvimarina pelagi HTCC2506 | ZP_01439214.1 |
| Gallus gallus | XP_421428.2 |
| Gemmata obscuriglobus UQM 2246 | ZP_02733982.1 |
| Gibberella zeae PH-1 | XP_381905.1 |
| Gibberella zeae PH-1 | XP_388061.1 |
| Hahella chejuensis KCTC 2396 | YP_434355.1 |
| Haloarcula marismortui ATCC 43049 | YP_136982.1 |
| Herpetosiphon aurantiacus ATCC 23779 | YP_001545265.1 |
| Hoeflea phototrophica DFL-43 | ZP_02164890.1 |
| Hoeflea phototrophica DFL-43 | ZP_02166029.1 |
| Homo sapiens | NP_653182.1 |
| Jannaschia sp. CCS1 | YP_509471.1 |
| Kordia algicida OT-1 | ZP_02163297.1 |
| Lysinibacillus sphaericus C3-41 | ACA37747.1 |
| Macaca mulatta | XP_001094065.1 |
| Maricaulis maris MCS10 | YP_755529.1 |
| Marine actinobacterium PHSC20C1 | ZP_01130534.1 |
| Marinobacter algicola DG893 | ZP_01892319.1 |
| Marinobacter aquaeolei VT8 | YP_959407.1 |
| Marinobacter sp. ELB17 | ZP_01736899.1 |
| Marinomonas sp. MED121 | ZP_01074684.1 |
| Marinomonas sp. MWYL1 | YP_001338929.1 |
| Mesorhizobium loti MAFF303099 | NP_104961.1 |
| Mesorhizobium loti MAFF303099 | NP_107249.1 |
| Mesorhizobium sp. BNC1 | YP_674147.1 |
| Methylobacterium nodulans ORS 2060 | ZP_02121094.1 |
| Methylobacterium nodulans ORS 2060 | ZP_02121102.1 |
| Methylobacterium sp. 4-46 | ZP_01847185.1 |
| Methylobacterium sp. 4-46 | ZP_01850691.1 |
| Microscilla marina ATCC 23134 | ZP_01688824.1 |
| Microscilla marina ATCC 23134 | ZP_01694596.1 |
| Monodelphis domestica | XP_001377162.1 |
| Mus musculus | AAH04753.1 |
| Mus musculus | EDL36543.1 |
| Mus musculus | NP_080314.1 |
| Myxococcus xanthus DK 1622 | YP_634330.1 |
| Nematostella vectensis | XP_001617584.1 |
| Nematostella vectensis | XP_001618064.1 |
| Nematostella vectensis | XP_001622857.1 |
| Nematostella vectensis | XP_001627872.1 |
| Neosartorya fischeri NRRL 181 | XP_001266333.1 |
| Oceanibulbus indolifex HEL-45 | ZP_02154826.1 |
| Oceanibulbus indolifex HEL-45 | ZP_02154828.1 |
| Oceanicola granulosus HTCC2516 | ZP_01156155.1 |
| Oceanicola granulosus HTCC2516 | ZP_01156156.1 |
| Ochrobactrum anthropi ATCC 49188 | YP_001368999.1 |
| Ochrobactrum anthropi ATCC 49188 | YP_001369660.1 |
| Ornithorhynchus anatinus | XP_001517024.1 |
| Pan troglodytes | XP_509980.2 |
| Paracoccus denitrificans PD1222 | ZP_00628393.1 |
| Paracoccus denitrificans PD1222 | ZP_00629761.1 |
| Paracoccus denitrificans PD1222 | ZP_00631581.1 |
| Paracoccus denitrificans PD1222 | ZP_00632519.1 |
| Phaeobacter gallaeciensis 2.10 | ZP_02149065.1 |
| Phaeobacter gallaeciensis 2.10 | ZP_02149767.1 |
| Phaeobacter gallaeciensis BS107 | ZP_02145678.1 |
| Phaeobacter gallaeciensis BS107 | ZP_02146598.1 |
| Phaeosphaeria nodorum SN15 | EAT90478.1 |
| Photorhabdus luminescens subsp. laumondii TTO1 | NP_929500.1 |
| Plesiocystis pacifica SIR-1 | ZP_01909430.1 |
| Plesiocystis pacifica SIR-1 | ZP_01909435.1 |
| Pseudoalteromonas tunicata D2 | ZP_01134861.1 |
| Pseudomonas aeruginosa | ABS82393.2 |
| Pseudomonas aeruginosa 2192 | ZP_00976473.1 |
| Pseudomonas aeruginosa C3719 | EAZ54528.1 |
| Pseudomonas aeruginosa C3719 | ZP_00970302.1 |
| Pseudomonas aeruginosa C3719 | ZP_00970345.1 |
| Pseudomonas aeruginosa PA7 | YP_001349476.1 |
| Pseudomonas aeruginosa PA7 | YP_001349491.1 |
| Pseudomonas aeruginosa PAO1 | NP_249946.1 |
| Pseudomonas aeruginosa PAO1 | NP_249959.1 |
| Pseudomonas aeruginosa UCBPP-PA14 | YP_791977.1 |
| Pseudomonas aeruginosa UCBPP-PA14 | YP_791990.1 |
| Pseudomonas chlororaphis | AAB00331.1 |
| Pseudomonas entomophila L48 | YP_609530.1 |
| Pseudomonas fluorescens Pf-5 | YP_258541.1 |
| Pseudomonas fluorescens PfO-1 | YP_348040.1 |
| Pseudomonas putida F1 | YP_001266630.1 |
| Pseudomonas putida GB-1 | YP_001670359.1 |
| Pseudomonas putida KT2440 | NP_743418.1 |
| Pseudomonas putida W619 | ZP_01639325.1 |
| Psychrobacter cryohalolentis K5 | YP_580484.1 |
| Psychroflexus torquis ATCC 700755 | ZP_01254321.1 |
| Psychromonas ingrahamii 37 | YP_943461.1 |
| Rattus norvegicus | NP_001101501.1 |
| Reinekea sp. MED297 | ZP_01112820.1 |
| Renibacterium salmoninarum ATCC 33209 | YP_001624965.1 |
| Rhizobium etli CFN 42 | YP_468000.1 |
| Rhizobium etli CFN 42 | YP_473008.1 |
| Rhizobium leguminosarum bv. trifolii WSM1325 | ZP_02291775.1 |
| Rhizobium leguminosarum bv. trifolii WSM1325 | ZP_02296079.1 |
| Rhizobium leguminosarum bv. trifolii WSM2304 | ZP_02854524.1 |
| Rhizobium leguminosarum bv. trifolii WSM2304 | ZP_02855897.1 |
| Rhizobium leguminosarum bv. trifolii WSM2304 | ZP_02857335.1 |
| Rhizobium leguminosarum bv. trifolii WSM2304 | ZP_02859145.1 |
| Rhizobium leguminosarum bv. trifolii WSM2304 | ZP_02859159.1 |
| Rhizobium leguminosarum bv. viciae 3841 | YP_765040.1 |
| Rhizobium leguminosarum bv. viciae 3841 | YP_766088.1 |
| Rhodobacter sphaeroides 2.4.1 | YP_355023.1 |
| Rhodobacter sphaeroides ATCC 17029 | YP_001045036.1 |
| Rhodobacterales bacterium HTCC2150 | ZP_01741305.1 |
| Rhodococcus sp. RHA1 | YP_704510.1 |
| Rhodococcus sp. RHA1 | YP_705227.1 |
| Rhodopirellula baltica SH 1 | NP_864728.1 |
| Rhodospirillum rubrum ATCC 11170 | YP_427152.1 |
| Robiginitalea biformata HTCC2501 | ZP_01122088.1 |
| Roseiflexus castenholzii DSM 13941 | YP_001430385.1 |
| Roseiflexus sp. RS-1 | YP_001275012.1 |
| Roseobacter sp. AzwK-3b | ZP_01902703.1 |
| Roseobacter sp. MED193 | ZP_01056457.1 |
| Roseobacter sp. MED193 | ZP_01057809.1 |
| Roseobacter sp. SK209-2-6 | ZP_01754446.1 |
| Roseobacter sp. SK209-2-6 | ZP_01755333.1 |
| Roseovarius nubinhibens ISM | ZP_00959811.1 |
| Roseovarius sp. 217 | ZP_01038422.1 |
| Roseovarius sp. 217 | ZP_01038424.1 |
| Roseovarius sp. TM1035 | ZP_01880611.1 |
| Roseovarius sp. TM1035 | ZP_01880616.1 |
| Rubrobacter xylanophilus DSM 9941 | YP_644995.1 |
| Rubrobacter xylanophilus DSM 9941 | YP_645001.1 |
| Saccharopolyspora erythraea NRRL 2338 | YP_001105452.1 |
| Serratia proteamaculans 568 | YP_001478071.1 |
| Shewanella amazonensis SB2B | YP_927407.1 |
| Shewanella amazonensis SB2B | YP_927409.1 |
| Shewanella benthica KT99 | ZP_02159784.1 |
| Shewanella halifaxensis HAW-EB4 | YP_001674769.1 |
| Shewanella halifaxensis HAW-EB4 | YP_001674771.1 |
| Shewanella loihica PV-4 | YP_001094486.1 |
| Shewanella loihica PV-4 | YP_001094488.1 |
| Shewanella pealeana ATCC 700345 | YP_001501562.1 |
| Shewanella pealeana ATCC 700345 | YP_001501564.1 |
| Shewanella sediminis HAW-EB3 | YP_001473500.1 |
| Shewanella sediminis HAW-EB3 | YP_001473502.1 |
| Shewanella woodyi ATCC 51908 | ZP_01541661.1 |
| Shewanella woodyi ATCC 51908 | ZP_01541663.1 |
| Silicibacter pomeroyi DSS-3 | YP_165095.1 |
| Silicibacter pomeroyi DSS-3 | YP_165867.1 |
| Silicibacter sp. TM1040 | YP_611435.1 |
| Silicibacter sp. TM1040 | YP_611437.1 |
| Sinorhizobium medicae WSM419 | YP_001312606.1 |
| Sinorhizobium medicae WSM419 | YP_001312608.1 |
| Sinorhizobium meliloti 1021 | NP_436798.1 |
| Sinorhizobium meliloti 1021 | NP_436800.1 |
| Solibacter usitatus Ellin6076 | YP_827786.1 |
| Stappia aggregata IAM 12614 | ZP_01549009.1 |
| Stappia aggregata IAM 12614 | ZP_01549011.1 |
| Streptomyces avermitilis MA-4680 | NP_822906.1 |
| Streptomyces coelicolor A3(2) | NP_630391.1 |
| Strongylocentrotus purpuratus | XP_787917.2 |
| Strongylocentrotus purpuratus | XP_790644.1 |
| Strongylocentrotus purpuratus | XP_791727.1 |
| Tetraodon nigroviridis | CAG03606.1 |
| Trypanosoma cruzi strain CL Brener | XP_803162.1 |
| Trypanosoma cruzi strain CL Brener | XP_811287.1 |
| Verminephrobacter eiseniae EF01-2 | YP_995838.1 |
| Vibrio alginolyticus 12G01 | ZP_01259188.1 |
| Vibrio parahaemolyticus AQ3810 | ZP_01989291.1 |
| Vibrio parahaemolyticus RIMD 2210633 | NP_797709.1 |
| Vibrio sp. Ex25 | ZP_01475805.1 |
| Xanthomonas axonopodis pv. citri str. 306 | NP_642548.1 |
| Xanthomonas axonopodis pv. citri str. 306 | NP_642865.1 |
| Xanthomonas campestris pv. campestris str. ATCC 33913 | NP_637769.1 |
| Xanthomonas campestris pv. vesicatoria str. 85-10 | YP_364479.1 |
| Xanthomonas oryzae pv. oryzae KACC10331 | YP_201780.1 |
| Xanthomonas oryzae pv. oryzae MAFF 311018 | YP_452012.1 |
| Xanthomonas oryzae pv. oryzicola BLS256 | ZP_02242836.1 |
| Xenopus tropicalis | NP_001008128.1 |
|  |  |

**(B)**

| alpha proteobacterium BAL199 | ZP_02190899.1 |
| --- | --- |
| alpha proteobacterium BAL199 | ZP_02190900.1 |
| alpha proteobacterium BAL199 | ZP_02190901.1 |
| Acidiphilium cryptum JF-5 | YP_001233471.1 |
| Acidovorax avenae subsp. citrulli AAC00-1 | YP_972753.1 |
| Acidovorax sp. JS42 | YP_988029.1 |
| Acinetobacter baumannii | CAM86110.1 |
| Acinetobacter baumannii | YP_001706707.1 |
| Acinetobacter baumannii ATCC 17978 | YP_001085319.1 |
| Agrobacterium tumefaciens str. C58 | NP_356385.2 |
| Alkaliphilus metalliredigens QYMF | YP_001322515.1 |
| Alkaliphilus oremlandii OhILAs | YP_001512294.1 |
| Arthrobacter aurescens TC1 | YP_949827.1 |
| Arthrobacter chlorophenolicus A6 | ZP_02838488.1 |
| Arthrobacter sp. FB24 | YP_833130.1 |
| Azotobacter vinelandii AvOP | ZP_00416954.1 |
| Bacillus pumilus SAFR-032 | YP_001488870.1 |
| Bacillus sp. B14905 | ZP_01724089.1 |
| Blastopirellula marina DSM 3645 | ZP_01088976.1 |
| Bradyrhizobium japonicum USDA 110 | NP_772283.1 |
| Bradyrhizobium sp. BTAi1 | YP_001242578.1 |
| Bradyrhizobium sp. ORS278 | YP_001203479.1 |
| Brevibacterium linens BL2 | ZP_00378489.1 |
| Brucella suis 1330 | NP_698373.1 |
| Burkholderia ambifaria AMMD | YP_774279.1 |
| Burkholderia ambifaria AMMD | YP_778332.1 |
| Burkholderia ambifaria MC40-6 | ZP_01554096.1 |
| Burkholderia ambifaria MC40-6 | ZP_01554793.1 |
| Burkholderia cenocepacia AU 1054 | YP_621617.1 |
| Burkholderia cenocepacia AU 1054 | YP_625928.1 |
| Burkholderia cenocepacia MC0-3 | ZP_01559819.1 |
| Burkholderia cenocepacia MC0-3 | ZP_01567029.1 |
| Burkholderia cenocepacia PC184 | ZP_00980256.1 |
| Burkholderia dolosa AUO158 | ZP_00987078.1 |
| Burkholderia mallei ATCC 23344 | YP_103786.1 |
| Burkholderia multivorans ATCC 17616 | YP_001579114.1 |
| Burkholderia multivorans ATCC 17616 | YP_001585583.1 |
| Burkholderia oklahomensis C6786 | ZP_02362170.1 |
| Burkholderia oklahomensis EO147 | ZP_02354954.1 |
| Burkholderia phymatum STM815 | ZP_01503935.1 |
| Burkholderia phytofirmans PsJN | ZP_01511279.1 |
| Burkholderia pseudomallei 14 | ZP_02410470.1 |
| Burkholderia pseudomallei 305 | ZP_01767946.1 |
| Burkholderia pseudomallei 668 | YP_001058201.1 |
| Burkholderia pseudomallei 7894 | ZP_02480788.1 |
| Burkholderia pseudomallei 9 | ZP_02454772.1 |
| Burkholderia pseudomallei 91 | ZP_02453357.1 |
| Burkholderia pseudomallei B7210 | ZP_02470361.1 |
| Burkholderia pseudomallei BCC215 | ZP_02505200.1 |
| Burkholderia pseudomallei K96243 | YP_107703.1 |
| Burkholderia sp. 383 | YP_369930.1 |
| Burkholderia sp. 383 | YP_372329.1 |
| Burkholderia sp. 383 | YP_372329.1 |
| Burkholderia thailandensis E264 | YP_441503.1 |
| Burkholderia thailandensis MSMB43 | ZP_02462803.1 |
| Burkholderia thailandensis TXDOH | ZP_02373147.1 |
| Burkholderia ubonensis Bu | ZP_02378245.1 |
| Burkholderia vietnamiensis G4 | YP_001120274.1 |
| Burkholderia xenovorans LB400 | YP_557701.1 |
| Caldicellulosiruptor saccharolyticus DSM 8903 | YP_001179478.1 |
| Campylobacter fetus subsp. fetus 82-40 | YP_892632.1 |
| Candida albicans WO-1* | CAWG_05038 |
| Candida albicans SC5314 | XP_719180.1 |
| Candida dubliniensis* | Cd63680 |
| Candida glabrata | XP_447736.1 |
| Candida lusitaniae* | CLUG_01317 |
| Candida parapsilosis* | CPAG_03462 |
| Candida tropicalis | CTRG_02110 |
| Chloroflexus aurantiacus J-10-fl | YP_001636460.1 |
| Chromobacterium violaceum ATCC 12472 | NP_900601.1 |
| Chromobacterium violaceum ATCC 12472 | NP_902333.1 |
| Clostridium acetobutylicum ATCC 824 | NP_350036.1 |
| Clostridium beijerinckii NCIMB 8052 | YP_001310573.1 |
| Clostridium bolteae ATCC BAA-613 | ZP_02082878.1 |
| Clostridium difficile 630 | YP_001089550.1 |
| Clostridium difficile QCD-32g58 | ZP_01802492.1 |
| Clostridium difficile QCD-63q42 | ZP_02747610.1 |
| Comamonas testosteroni KF-1 | ZP_01522720.1 |
| Corynebacterium glutamicum ATCC 13032 | NP_601940.2 |
| Corynebacterium glutamicum R | YP_001139565.1 |
| Cyanothece sp. CCY0110 | ZP_01730452.1 |
| Debaryomyces hansenii CBS767 | XP_457731.1 |
| Delftia acidovorans SPH-1 | YP_001561623.1 |
| Delftia acidovorans SPH-1 | YP_001566889.1 |
| Desulfitobacterium hafniense Y51 | YP_517649.1 |
| Desulfuromonas acetoxidans DSM 684 | ZP_01312422.1 |
| Dinoroseobacter shibae DFL 12 | YP_001534079.1 |
| Erwinia carotovora subsp. atroseptica SCRI1043 | YP_050790.1 |
| Erwinia carotovora subsp. atroseptica SCRI1043 | YP_051101.1 |
| Erwinia chrysanthemi | CAC59746.1 |
| Frankia alni ACN14a | YP_712625.1 |
| Frankia sp. CcI3 | YP_481067.1 |
| Frankia sp. EAN1pec | YP_001506330.1 |
| gamma proteobacterium KT 71 | ZP_01104816.1 |
| Gluconacetobacter diazotrophicus PAl 5 | YP_001602674.1 |
| Halobacterium sp. NRC-1 | NP_279428.1 |
| Halobacterium sp. NRC-1 | NP_395881.1 |
| Herpetosiphon aurantiacus ATCC 23779 | YP_001545008.1 |
| Hoeflea phototrophica DFL-43 | ZP_02166138.1 |
| Janibacter sp. HTCC2649 | ZP_00997320.1 |
| Jannaschia sp. CCS1 | YP_509080.1 |
| Kineococcus radiotolerans SRS30216 | YP_001363801.1 |
| Kluyveromyces lactis | XP_454466.1 |
| Lyngbya sp. PCC 8106 | ZP_01624285.1 |
| Malassezia globosa CBS 7966 | XP_001731613.1 |
| Methylibium petroleiphilum PM1 | YP_001019384.1 |
| Methylobacterium populi BJ001 | ZP_02199989.1 |
| Mycobacterium ulcerans Agy99 | YP_906542.1 |
| Natronomonas pharaonis DSM 2160 | YP_326261.1 |
| Nocardioides sp. JS614 | YP_923502.1 |
| Ochrobactrum anthropi ATCC 49188 | YP_001370364.1 |
| Paracoccus denitrificans PD1222 | ZP_00630713.1 |
| Photorhabdus luminescens subsp. laumondii TTO1 | NP_929528.1 |
| Pichia guilliermondii ATCC 6260 | XP_001487811.1 |
| Pichia stipitis CBS 6054 | XP_001382977.2 |
| Polaromonas naphthalenivorans CJ2 | YP_980644.1 |
| Polaromonas sp. JS666 | YP_551032.1 |
| Pseudomonas aeruginosa C3719 | ZP_00970127.1 |
| Pseudomonas aeruginosa PA7 | YP_001349371.1 |
| Pseudomonas aeruginosa PACS2 | ZP_01364735.1 |
| Pseudomonas aeruginosa PAO1 | NP_250058.1 |
| Pseudomonas aeruginosa PAO1 | NP_250595.1 |
| Pseudomonas aeruginosa UCBPP-PA14 | YP_791879.1 |
| Pseudomonas fluorescens Pf-5 | YP_259395.1 |
| Pseudomonas putida W619 | ZP_01641735.1 |
| Pseudomonas syringae pv. phaseolicola 1448A | YP_274694.1 |
| Pseudomonas syringae pv. syringae B728a | YP_235440.1 |
| Pseudomonas syringae pv. tomato str. DC3000 | NP_792432.1 |
| Ralstonia eutropha JMP134 | YP_295060.1 |
| Reinekea sp. MED297 | ZP_01116734.1 |
| Rhizobium etli CFN 42 | YP_470984.1 |
| Rhizobium leguminosarum bv. trifolii WSM1325 | ZP_02291652.1 |
| Rhizobium leguminosarum bv. trifolii WSM2304 | ZP_02855132.1 |
| Rhizobium leguminosarum bv. viciae 3841 | YP_769590.1 |
| Rhodobacterales bacterium HTCC2654 | ZP_01012764.1 |
| Rhodoferax ferrireducens T118 | YP_524962.1 |
| Rhodopirellula baltica SH 1 | NP_869724.1 |
| Rhodopirellula baltica SH 1 | NP_870014.1 |
| Rhodopseudomonas palustris BisB18 | YP_533219.1 |
| Rhodopseudomonas palustris CGA009 | NP_948521.1 |
| Rhodopseudomonas palustris TIE-1 | ZP_02299787.1 |
| Roseobacter sp. CCS2 | ZP_01751425.1 |
| Roseovarius sp. 217 | ZP_01036042.1 |
| Saccharomyces cerevisiae | NP_011894.1 |
| Saccharomyces cerevisiae YJM789 | EDN62266.1 |
| Saccharopolyspora erythraea NRRL 2338 | YP_001107033.1 |
| Sagittula stellata E-37 | ZP_01746081.1 |
| Salinispora arenicola CNS-205 | YP_001535578.1 |
| Salinispora tropica CNB-440 | YP_001157573.1 |
| Salinispora tropica CNB-440 | YP_001157895.1 |
| Schizosaccharomyces pombe 972h- | NP_001018772.1 |
| Shewanella baltica OS155 | YP_001050311.1 |
| Sinorhizobium medicae WSM419 | YP_001327601.1 |
| Sinorhizobium medicae WSM419 | YP_001328320.1 |
| Sinorhizobium meliloti 1021 | NP_386147.1 |
| Sinorhizobium meliloti 1021 | NP_386874.1 |
| Sodalis glossinidius str. 'morsitans' | YP_455775.1 |
| Stappia aggregata IAM 12614 | ZP_01548236.1 |
| Stenotrophomonas maltophilia R551-3 | ZP_01644219.1 |
| Stenotrophomonas maltophilia R551-3 | ZP_01644220.1 |
| Synechocystis sp. PCC 6803 | NP_440471.1 |
| Trichodesmium erythraeum IMS101 | YP_720917.1 |
| Ustilago maydis 521 | XP_758000.1 |
| Vanderwaltozyma polyspora DSM 70294 | XP_001644231.1 |
| Xanthobacter autotrophicus Py2 | YP_001416232.1 |
| Xanthomonas axonopodis pv. citri str. 306 | NP_643221.1 |
| Xanthomonas axonopodis pv. citri str. 306 | NP_643222.1 |
| Xanthomonas campestris pv. campestris | CAP50768.1 |
| Xanthomonas campestris pv. campestris str. ATCC 33913 | NP_638090.1 |
| Xanthomonas campestris pv. campestris str. ATCC 33913 | NP_638091.1 |
| Xanthomonas campestris pv. vesicatoria str. 85-10 | YP_364788.1 |
| Xanthomonas campestris pv. vesicatoria str. 85-10 | YP_364789.1 |
| Xanthomonas oryzae pv. oryzae KACC10331 | YP_200069.1 |
| Xanthomonas oryzae pv. oryzae KACC10331 | YP_200071.1 |
| Xanthomonas oryzae pv. oryzae MAFF 311018 | YP_450343.1 |
| Xanthomonas oryzae pv. oryzae MAFF 311018 | YP_450345.1 |
| Xanthomonas oryzae pv. oryzicola BLS256 | ZP_02242476.1 |
| Xanthomonas oryzae pv. oryzicola BLS256 | ZP_02242477.1 |
| Yersinia frederiksenii ATCC 33641 | ZP_00828808.1 |
| Zymomonas mobilis subsp. mobilis ZM4 | YP_163535.1 |
| Zymomonas mobilis subsp. mobilis ZM4 | YP_163680.1 |
