# Supplementary figures and images for: Evidence of recent interkingdom horizontal gene transfer between bacteria and Candida parapsilosis
Source: BMC Evol Biol. 2008 Jun 24;8:181. doi: 10.1186/1471-2148-8-181 (PMC2459174; doi:10.1186/1471-2148-8-181)

(1)

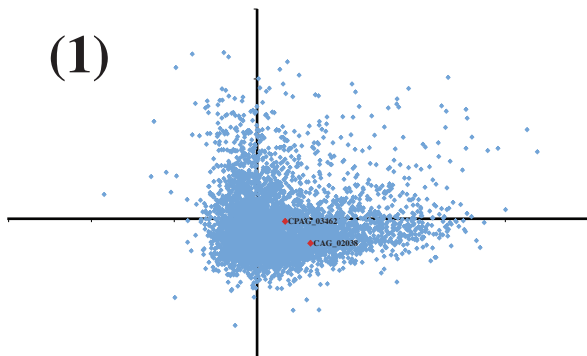

(2)

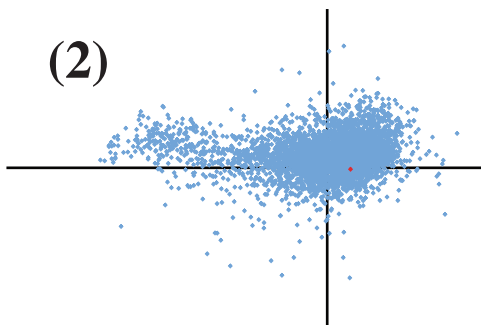

(3)

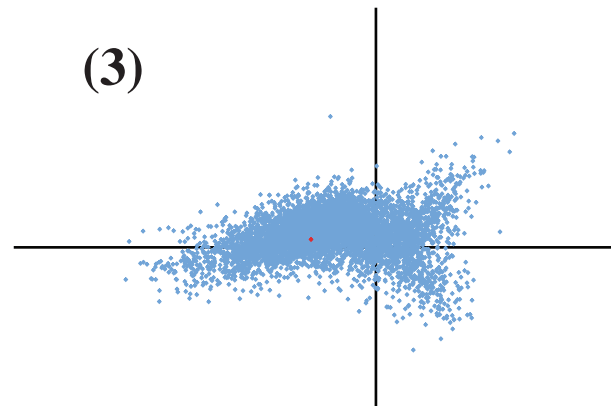

(4)

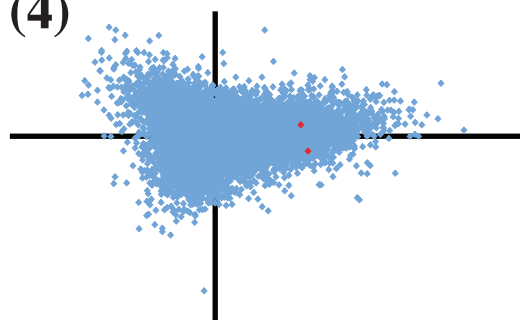

(5)

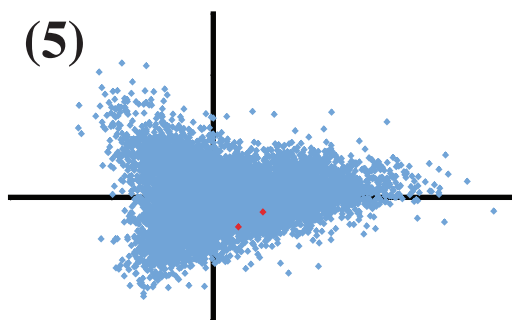

(6)

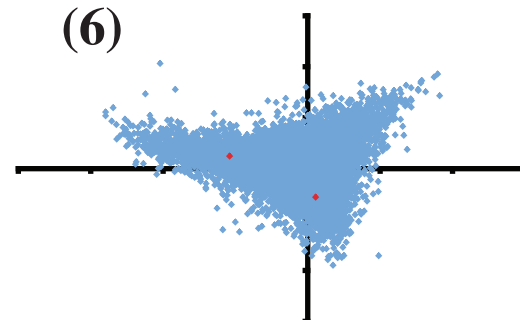

(7)

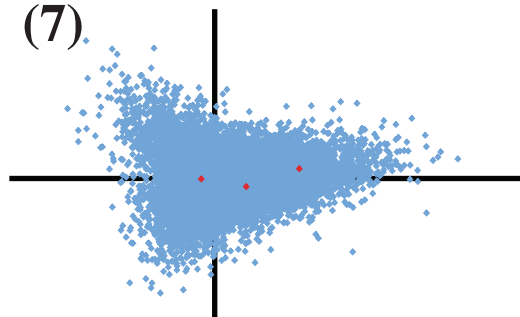

(8)

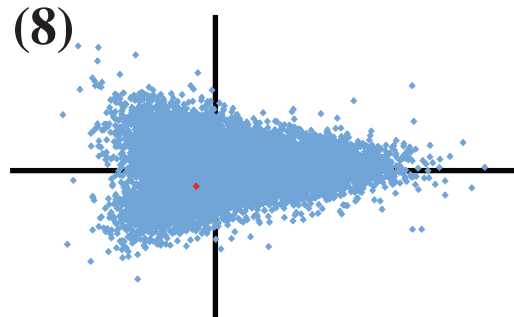

(9)

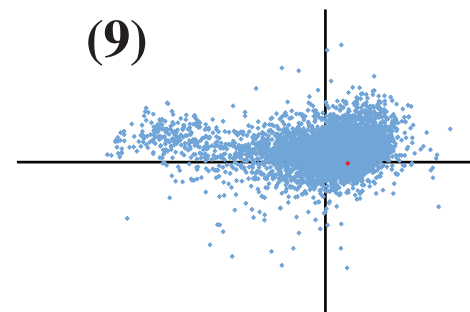

Supplement: Additional file 4 — Correspondence analysis of codon usage. Correspondence analysis of codon usage in the C. parapsilosis (1), U. maydis (2), M. globosa (3), A. flavus (4), A. niger (5), G. zeae (6), A. oryzae (7), P. nodorum (8), and S. pombe (9) genomes. Transferred genes are highlighted. All have a codon usage similar to the rest of their genomes which is unsurprising as transferred genes have been shown to ameliorate their codon usage to their hosts [79]. [file 1471-2148-8-181-S4.pdf]
